# The Effects of Four-Week Multivitamin Supplementation on Mood in Healthy Older Women: A Randomized Controlled Trial

**DOI:** 10.1155/2016/3092828

**Published:** 2016-11-15

**Authors:** Helen Macpherson, Renee Rowsell, Katherine H. M. Cox, Jeffery Reddan, Denny Meyer, Andrew Scholey, Andrew Pipingas

**Affiliations:** ^1^Institute for Physical Activity and Nutrition, School of Exercise and Nutrition Sciences, Deakin University, Melbourne, VIC, Australia; ^2^Centre for Human Psychopharmacology, Swinburne University, Melbourne, VIC, Australia; ^3^Faculty of Health, Arts and Design, Swinburne University, Melbourne, VIC, Australia

## Abstract

*Objective*. Nutritional deficiencies have been associated with cognitive decline and mood disturbances. Vitamin intake can influence mood and randomized controlled trials have demonstrated that multivitamin supplements are capable of reducing mild symptoms of mood dysfunction. However, few studies have focussed on healthy older women.* Methods*. This study investigated the effects of four weeks' multivitamin supplementation on mood in 76 healthy women aged 50–75 years. Mood was assessed before and after intervention in the laboratory using measures of current mood and retrospective experiences of mood over the past week or longer. Mobile phones were used to assess changes in real-time mood ratings, twice weekly in the home.* Results*. There were no multivitamin-related benefits identified for measures of current mood or reflections of recent mood when measured in the laboratory. In-home assessments, where mood was rated several hours after dose, revealed multivitamin supplementation improved ratings of stress, with a trend to reduce mental fatigue.* Conclusions*. Over four weeks, subtle changes to stress produced by multivitamin supplementation in healthy older women may not be detected when only pre- and posttreatment mood is captured. In-home mobile phone-based assessments may be more sensitive to the effects of nutritional interventions compared to traditional in-laboratory assessments.

## 1. Introduction

Vitamin insufficiency is common amongst older people [1, 2] and can lead to detriments to neurological function [3]. For instance, suboptimal intake of vitamins and minerals including folate, vitamin B6, vitamin B12, and zinc has been associated with mild psychiatric symptoms [4] and mood disorders in older people [5, 6]. In addition to psychiatric disturbances, deficiency of selected nutrients including folate and vitamin B12 has also been implicated in dementia [7]. Depression and anxiety represent significant risk factors for cognitive decline and dementia [8, 9]. Importantly, these dementia risk factors are modifiable and can serve as potential targets for intervention.

Multivitamins contain a range of B vitamins, as well as antioxidant vitamins and minerals which exert effects on the central nervous system including synthesis of catecholamine neurotransmitters and serotonin [10, 11]. The potential for multivitamins to modify mood has been demonstrated in a meta-analysis of eight randomized controlled trials (RCTs) which indicated that multivitamin use over a period of ≥4 weeks can reduce mild symptoms of mood dysfunction in healthy people, free from clinical mood disorders [12]. Only one of these trials focussed exclusively on people over the age of 50 years [13], demonstrating that, in men aged 50–70 years, eight weeks' supplementation with a multivitamin mineral and herbal (MVMH) formula improved ratings of current mood as well as reflections of mood over the preceding week.

It has been suggested that micronutrient interventions should show larger effects on mood when measured using near-to-real-time assessments, which permit mood to be tracked on a momentary basis, than when assessed by measures which rate experiences of mood over the past week or month [14]. This may be due to effects of micronutrients on neurotransmitters, as neurotransmitter and hormonal influences have more direct associations with immediate mood responses compared to delayed reflections on mood based on a longer time period [14, 15]. Our recent examination of acute effects of multivitamin supplementation conducted in women aged 50–75 years demonstrated benefits to general mood and perceived stress one to two hours after MVMH supplementation [16]. These findings indicate that, in older people, mood improvements can arise in the hours following multivitamin supplementation. Therefore a supplementation period of several months may not be necessary to detect mood improvements in older people due to multivitamin supplementation. For this reason there is a need to identify mood measures that employ a suitable time scale to best detect mood changes in shorter term intervention trials.

Ecological momentary assessment (EMA) is a methodology which enables an individual to report on symptoms, mood, and other behavioural parameters close in time to experience, and these reports are obtained many times over the course of a study [17]. EMA can provide real-time measures of mood and affords greater assessment of dynamic processes (i.e., change in mood over time) than laboratory based assessments [18]. There is emerging evidence that EMA methods may be particularly sensitive to the mood enhancing effects of multivitamin supplementation. In healthy young people, four weeks' multivitamin supplementation resulted in increased mental stamina, physical stamina, and concentration when mood was rated weekly on mobile phone devices [19] and four months' multivitamin supplementation improved ratings of current stress, physical fatigue, and anxiety using similar methodology [20]. By contrast, there were no benefits of multivitamins to mood or general well-being when assessments were conducted in a laboratory setting using traditional pretreatment/posttreatment mood measurement [20]. While these studies measured mood at multiple time points outside of the laboratory setting, the rates of mood changes were not examined in a continuous manner. To date, no RCTs have utilised EMA methodology to investigate the time course of multivitamin-related mood changes in older people.

This investigation extends our examination of acute effects of multivitamin supplements in older women [16] and reports the results of a longer four-week supplementation period in the same participant sample, using the same MVMH preparation. Our analysis from the acute time point identified immediate mood improvements, particularly improvements to stress, one to two hours after MVMH intake [16]. In the current study mood was assessed in the laboratory using standard mood measures which rated retrospective experiences of mood over the past week or longer, as well as using assessments which rated current experiences of mood. We set out to examine whether ratings of current mood, including energy levels, alertness, stress, and anxiety, would be more sensitive to the effects of four weeks' multivitamin mineral and herbal (MVMH) supplementation compared to retrospective mood measures. A further aim of this study was to assess real-time mood changes due to the MVMH supplements over the four-week period. To achieve this purpose, mood was rated in the home, twice weekly using mobile phones.

## 2. Methods

### 2.1. Trial Design and Randomization

This study followed a double-blind, placebo-controlled, parallel group, randomized design. Participants were allocated to receive either the MVMH formula or a placebo matched for appearance, smell, and taste. Participants were randomized in blocks of 4, with a ratio of 1 : 1 using a computer generated sequence. Randomization was implemented by personnel not involved in the trial, using sequentially numbered treatments. The study protocol was approved by the Swinburne University of Technology Human Research Ethics Committee (SUHREC) and was carried out in accordance with the Declaration of Helsinki. All participants signed a consent form prior to enrolment in the trial. The trial is registered as the “Behavioural Effects of Multivitamin Supplements” study on the Australian New Zealand Clinical Trials Registry (ACTRN12613001087741). Data collection took place between November 2013 and July 2014.

### 2.2. Participants

The sample consisted of 76 community dwelling females aged 50–75 years (M = 63.6 years, SD = 6.4 years), who were not currently engaged in full-time employment. We focussed on this age range as previous work by our group has identified mood benefits due to multivitamin supplementation in men aged 50–70 years [13], but relevant research in older women is lacking. Participants were recruited from the community using an existing database and via newspaper and post advertisements. All participants were in good health, English speaking, nonsmokers, and free from diabetes, cardiovascular disease, dementia, stroke, and other neurological conditions. Further exclusion criteria included a history of head trauma, alcohol abuse, clinically diagnosed depression, anxiety, or other psychiatric disorders, and use of antidepressant medication, antianxiety medication, anticholinergic drugs, acetylcholinesterase inhibitors, or high-dose anticoagulants. Participants who received a score below 25 on the Mini Mental State Examination (MMSE) [21] were ineligible as this score may indicate the presence of cognitive decline. All participants were required to abstain from using vitamin E, multivitamins, vitamin B complex, ginkgo biloba, fish oils, and St John's Wort supplementation for 4 weeks preceding the first study visit and for the duration of the study.

### 2.3. Interventions

The MVMH supplement was Swisse Women's 50+ Ultivite (Australian Register of Therapeutic Goods ID: 187121). The study treatment was given orally in tablet form. Supplements were packaged in blister packs containing 7 supplements labelled with each day of the week. Each participant received an opaque box containing 5 blister packs (35 supplements). Participants were required to take one tablet daily with breakfast for 4 weeks (~30 supplements).  Table 1 lists the ingredients of the MVMH supplement. As shown in Table 1 the majority of the non-MVM ingredients are at subtherapeutic levels with the exceptions of ashwagandha, ginkgo, and grape seed. The placebo tablets contained starch and a small amount of riboflavin (2 mg) to give them a similar smell and colouration of the urine. Participants were required to abstain from the treatment on the day of posttreatment testing. Treatment compliance was determined using a daily tablet taking log and by counting remaining tablets at the posttreatment assessment.

### 2.4. Sample Size

A meta-analysis [12] has indicated that multivitamin formulas, with comparable doses of B vitamins to the current study, exert small to medium sized effects (standard mean difference = .29) on mood measures including the General Health Questionnaire. Power analysis was conducted using G^*∗*^Power 3.1.3. To have 80% chance of detecting an effect size of this magnitude (*F* = .15) in a two-armed study (multivitamin, placebo) with at least 3 time points it was determined that a total sample of 75 participants would be required (alpha level = .05).

### 2.5. Procedure

Potential participants were screened over the telephone to determine initial eligibility. Those who fulfilled the eligibility criteria were invited to attend the first session where additional screening and baseline and acute data (published elsewhere, [16]) were collected.

#### 2.5.1. Baseline Visit

Participants attended the baseline laboratory based assessment between 0900 and 1100 hours. On the day of the baseline visit participants were asked to refrain from caffeine ingestion and to consume their “usual” breakfast. The participant's breakfast was recorded and they were requested to consume the same breakfast on the day of the posttreatment study visit. At the baseline visit, participants provided informed consent and completed a medical health questionnaire and the MMSE prior to enrolment in the study. Participants completed baseline retrospective mood ratings and current mood ratings using a mobile phone device, as well as the cognitive assessments, prior to being randomized to receive the MVMH formula or placebo. Participants completed a brief food frequency questionnaire which assessed general intake of 29 different foods over the past 12 months. Daily serves of fruit and vegetable intake were scored on a 6-point Likert scale from 0 (none) to 5 (4 or more).

#### 2.5.2. Mood Assessments in the Home

Participants were provided with a mobile phone device and were instructed to rate their current mood using Visual Analogue Scales on two days each week for the four-week period. Participants were asked to ensure there were at least two days separating each mood report and that mood reports were completed after MVMH intake for that day. Participants were requested to complete the mood assessments at 1000 or 1500 hours and to ensure they completed the assessments at both times across the intervention period.

#### 2.5.3. Posttreatment Visit

Participants returned for their posttreatment appointment 4 weeks later at the same time of day as their baseline visit. All mood measures were repeated, with exception of the 1-hour postdose assessment.

### 2.6. Measures

#### 2.6.1. Ratings of Current Mood

The State-Trait Anxiety Inventory-State (STAI-S) assesses intensity of individual's current state of anxiety using 20 items [22]. Scores range from 20 to 80 with higher scores indicating greater anxiety. Participants also rated their current mood using scales presented on mobile phone devices both during the study visits and at home. The Bond Lader Visual Analogue Scales (VAS) [23] were used to assess feelings of alertness, contentedness, and calmness. Participants marked the position of their current subjective state on a horizontal line anchored at either end by adjective pairs (e.g., happy-sad). Each line was scored as the percentage of the total distance from the negative anchor, with higher scores indicating more positive mood states. The alertness, contentedness, and calmness subscales were calculated from 16 adjective pairs. Additional VAS measures were used to assess current levels of stress, anxiety, concentration, physical fatigue, and mental fatigue on lines with end-points labelled “Not at all” and “Extremely.” Each scale provided a single subjective score between 0 and 100, with lower scores indicative of more desirable mood states on the mood scales and higher energy levels on the fatigue scales. Higher scores on the concentration item were indicative of greater ability to concentrate.

#### 2.6.2. General Health Questionnaire

Participants completed a number of pen-and-paper measures designed for use in nonclinical samples. The General Health Questionnaire-28 (GHQ-28) [24] assesses general mild psychiatric symptoms experienced over the past week using 28 items relevant to health-related quality of life. In nonclinical samples, improved mood ratings have been observed on the GHQ following multivitamin supplementation of ≥28 days [12]. Scores on the GHQ-28 range from 0 to 84, with lower scores indicating better health-related quality of life. Participants completed the General Health Questionnaire (GHQ) at an additional 2-week time point in the home and returned the questionnaire via mail.

#### 2.6.3. Additional Retrospective Mood Measures

All other pen-and-paper measures were completed at baseline and posttreatment study visits only. The Hospital Anxiety and Depression Scale (HADS) [25] is a commonly used measure designed to screen for mood disorders in general (nonpsychiatric) medical outpatients. The HADS provides a brief measure of anxiety and depression experienced over the past week. Scores on each subscale range from 0 to 21, with higher scores indicating more severe anxiety or depression. The Perceived Stress Scale (PSS) [26] was used to measure the degree to which respondents viewed situations which occurred over the past month as stressful. Scores on the PSS range from 0 to 40 with higher scores indicating higher levels of perceived stress. The Chalder Fatigue Scale [27] was used to measure severity of symptoms relating to physical and mental fatigue experienced over the past week. The scale consists of 14 items. Scores range from 0 to 42, with higher scores indicating greater fatigue.

### 2.7. Analysis

Statistical analyses were carried out in SPSS version 22. Independent groups *t*-tests were used to examine baseline group difference in age, body mass index, and education. Multivariate analysis of variance (MANOVA) was used to examine baseline group differences in mood.

Education was controlled for when examining all mood outcomes; 2 (treatment: multivitamin, placebo) × 2 (time: baseline, posttreatment) repeated mixed methods MANCOVA was conducted for the VAS and STAI measures of current mood.

A 2 (treatment: multivitamin, placebo) × 3 (time: baseline, 2 weeks, and posttreatment) MANCOVA was used for the analysis of the subscales of the GHQ-28. To avoid multicollinearity the total GHQ score was analysed in a separate ANCOVA model. An additional 2 (treatment: multivitamin, placebo) × 2 (time: baseline, posttreatment) MANCOVA was conducted for the Chalder Fatigue Scale, HADS, and PSS.

Secondary analysis was conducted on data from the EMA mobile phone VAS assessments using logistic longitudinal multilevel modelling in HLM7. This analysis was conducted to examine the dynamic effects of the MVMH formula on mood over time. The VAS scores were highly clustered around two distinct values, indicating they were not suitable for transformation. As these values represented the default low and high scores in the mobile phone program, VAS scores were recoded in binary form as below the midpoint ≤50 or above the midpoint >50. The default scores refer to the scores obtained on the scale if the participant pressed the left button first (score of 24 out of 100) or right button first (score of 76 out of 100).

Approximately eight time points were included in the multilevel analysis for each participant, commencing at the baseline laboratory session and concluding at the final in-home assessment.

## 3. Results

**Figure 1:**
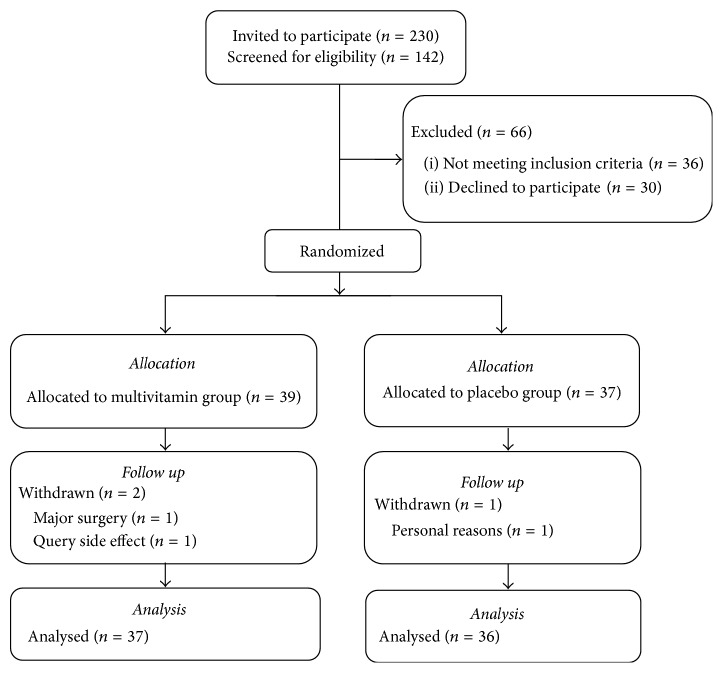
Recruitment and retention flowchart.

Participants were 39 women allocated to the multivitamin group and 37 allocated to placebo group. The participant recruitment flowchart is shown in Figure 1. Three participants withdrew during the course of the study leaving a total of 37 individuals in the multivitamin group and 36 in the placebo group (4% withdrawal rate).

Demographic details of the participant sample are shown in Table 2. Details of medication use have been reported elsewhere [16]. Independent *t*-tests indicated there were no significant group differences in participant age or body mass index (kg/m^2^); however participants assigned the MVMH treatment had completed significantly more years of education (*t*(74) = 2.19, *p* < .05). The majority of participants were either retired (46%) or working part time/casual hours (38%). The majority of participants reported consuming 2 serves of fruit per day (56% multivitamin group, 57% placebo group) and 3 serves of vegetables (49% multivitamin group, 33% placebo group). Chi square tests of independence indicated there were no interactions between treatment group and daily portions of fruit (*χ* ^2^(4) = 2.91, *p* = .57) or vegetable intake fruit (*χ* ^2^(4) = 4.22, *p* = .38). Average compliance for both groups was 99% (SD = 3), with no participants dropping below 89% adherence. On average participants completed 7 (SD = 1.2) mood assessments in the home using the mobile phone device. MANOVA analysis indicated at baseline there were no significant group differences between mood ratings on the GHQ measures (*F*(4,71) = .66,* Wilk's λ* = .96, and *p* = .62), visual analogue measures, and STAI (*F*(9,66) = .85,* Wilk's λ* = .90, and *p* = .58) or other retrospective mood measures (*F*(5,70) = 1.73,* Wilk's λ* = .89, and *p* = .14).

### 3.1. Laboratory Mood Assessments

Baseline and posttreatment mood scores are shown in Table 3. In terms of the effect of the MVMH formula on mood, there was no significant time × treatment interaction for the GHQ-28 total score (*F*(2,128) = .78, *p* = .46). A significant time × treatment interaction was identified for the GHQ-28 subscores (*F*(8,57) = 2.27,* Wilk's λ* = .76, and *p* = .04). However, the univariate time × treatment interaction was not significant for any of these measures in isolation. There were no significant time × treatment interactions for the Bond Lader VAS, additional VAS, and STAI ratings of current mood (*F*(9,60) = .97,* Wilk's λ* = .87, and *p* = .50). Similarly there were no significant time × treatment interactions for the other retrospective mood scales including the Chalder Fatigue total score and subscales, the HADS, and the PSS (*F*(5,65) = .94,* Wilk's λ* = .93, and *p* = .47).

### 3.2. EMA Assessments of Current Mood in the Home

**Figure 2:**
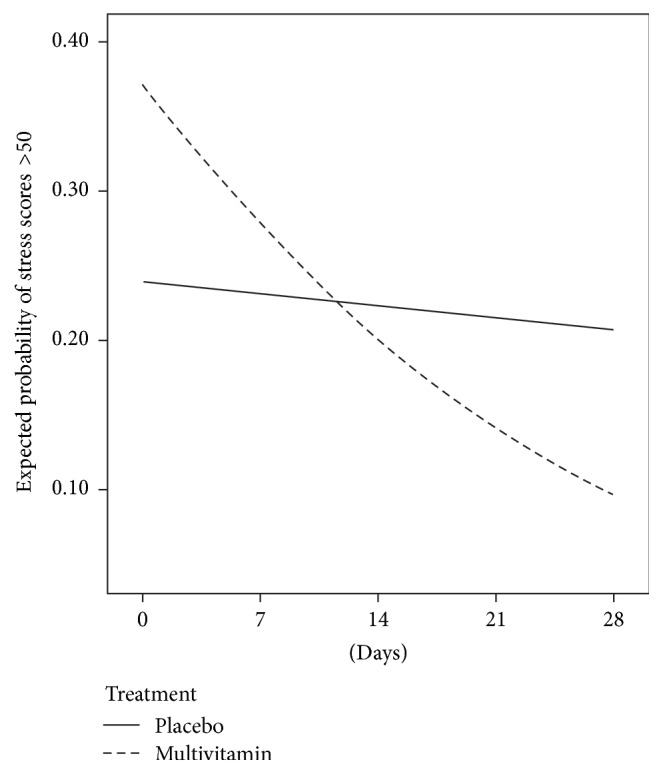
Change in the probability of stress ratings >50, assessed biweekly using mobile phone devices, commencing at baseline and concluding at the final in-home assessment for someone with 12 years of education.

**Figure 3:**
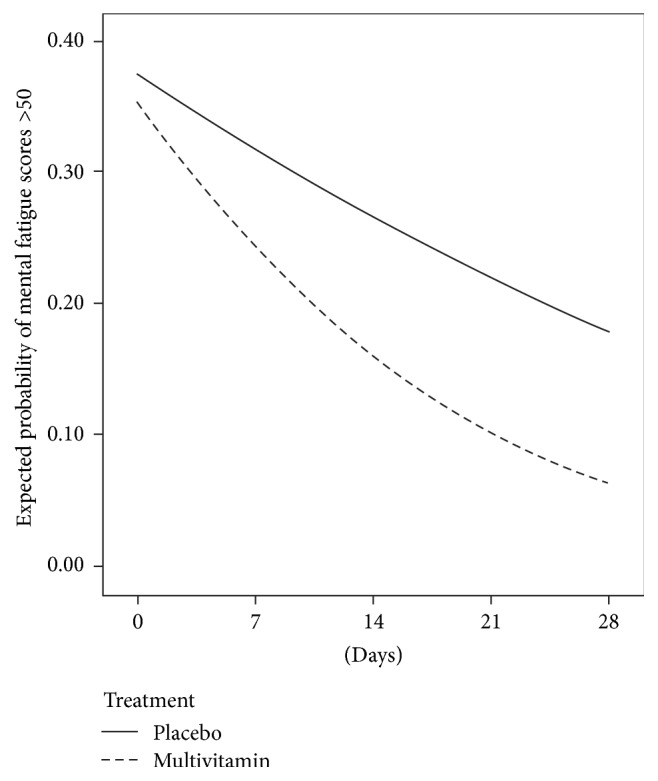
Change in the probability of mental fatigue ratings >50 assessed biweekly using mobile phone devices, commencing at baseline and concluding at the final in-home assessment for someone with 12 years of education.

In total 54% of mood ratings were completed in the morning, with 40% completed in the the afternoon and 6% in the evening. This was highly comparable for both treatment groups. Slope and odds ratio values for each group are shown in Table 4 while controlling for years of education. A significant interaction between time and treatment was identified for stress (*t*(71) = 2.82, *p* = .006) and so was a very nearly significant interaction effect for mental fatigue (*t*(71) = 1.957, *p* = .054). Odds ratios indicate the daily reduction in stress was on average 5.3% greater in the multivitamin group than the placebo and reduction in mental fatigue was on average 3.7% greater in the multivitamin group than in the placebo group. Changes in stress and mental fatigue ratings, concluding at the final in-home assessment, are shown in Figures 2 and 3, respectively.

## 4. Discussion

This study of healthy older women found no benefit of four weeks' MVMH supplementation to ratings of retrospective or current experiences of mood. However, improvements to stress and a trend for mental fatigue to be reduced were observed when mood was rated in the home on multiple occasions, using mobile phone devices. These results differ from trials which have demonstrated that a period of 4 weeks' multivitamin supplementation is sufficient to induce mood benefits to similar retrospective measures in healthy younger adults [28, 29]. However findings of mood benefits detected outside the laboratory are consistent with results of other researchers who have utilised comparable mobile phone based assessments [19, 20].

The present study did not identify multivitamin-related benefits to measures of current mood or retrospective mood, when rated in the laboratory. Improvements to both categories of mood ratings on several of the same measures included in the present study, including the GHQ and VAS mood scales, have previously been found to benefit from multivitamin supplementation in men aged 50–69 years, over a period of eight weeks, using a similar MVMH formulation [13]. These results suggest the mood measures used in the present study were suitable to detect mood changes due to the MVMH formula. Furthermore, our analysis from an acute time point in the same study identified immediate mood improvements, particularly improvements to stress, one to two hours after MVMH intake [16]. These findings indicate that the cohort under investigation was responsive to the MVMH treatment and this observation was confirmed in the EMA analysis of mobile phone data undertaken in the current investigation. When considered together the results of this study suggest that the four-week intervention duration may not have been sufficient to induce ongoing mood changes which could be detected in the laboratory when participants had been instructed to abstain from taking the MVMH formula.

There were no multivitamin-related improvements to current mood when measured in the laboratory; however a gradual reduction in stress and mental fatigue was observed in the home across repeated measurements. These results do need to be interpreted with a certain degree of caution as the data was required to be converted into binary form, due to clustering of scores. There are several factors which may have influenced differential home and laboratory results. Firstly, for this cohort the home environment is likely to be less stressful and thus less likely to mask any beneficial mood effects compared to the laboratory. An important consideration is that participants were required to abstain from taking the treatment on the day of the laboratory based follow-up assessments; however at-home biweekly assessments were completed after MVMH intake. Our prior analysis of acute effects of MVMH supplementation from this trial indicated that mood improvements, particularly improvements to stress, occurred 1-2 hours after dose, in the same participant sample, using the same MVMH preparation [16]. Similar immediate mood benefits of multivitamin supplements have been reported by others in younger cohorts [30, 31].

When considered along with previous observations of an acute mood benefit arising from multivitamin supplementation [16], results from the current study suggest that the mood enhancements captured by the EMA methodology may partially reflect more immediate effects of the MVMH formula induced by taking the supplement earlier on the same day as the mood ratings were completed. This interpretation would be consistent with the results of Pipingas et al. [20] who identified benefits to current ratings of mood only on days when participants had consumed the MVMH supplement. Our study extends the findings of Pipingas et al. [20] by demonstrating that multivitamin supplementation can lead to an ongoing reduction in stress and mental fatigue over time and not just when discrete trial end-points are considered. These findings indicate that multivitamin-related benefits to mood may reflect short term postintake mood improvements coupled with a cumulative effect which increases over time. While future studies wanting to solely focus on chronic effects of multivitamins should consider abstinence from treatment prior to posttreatment assessments, the time course and limits of both the acute and cumulative effects of multivitamin supplementation also merit further investigation.

There is evidence from a meta-analysis conducted by Long and Benton [12], which indicates that, across studies, multivitamins significantly improved both stress and mental fatigue facets of mood. This observation has been reported across time frames ranging from hours [16], weeks [28, 29], and through to months after dose [32]. In terms of a putative mechanism, vitamins B2 and B6 and niacin are necessary for amino acid metabolism required for the production of serotonin, a neurotransmitter important for mood regulation [10]. Additionally, vitamins B_12_ and folate and B_6_ are crucial for one-carbon metabolism, a process through which* S*-adenosylmethionine (SAMe) is formed. SAMe, the major methyl donor in the body is critical for the production of the neurotransmitters norepinephrine and dopamine, as well as serotonin [33]. Others have shown that three months' supplementation with a drink containing multivitamins and minerals increased levels of serum serotonin, with tryptophan levels increasing over six months [34]. Serum tryptophan levels have been demonstrated to be a useful biomarker to distinguish moderate and severe depression from healthy controls [35], indicating a relationship with mood regulation. Whether changes to levels of serotonin or tryptophan occurred in the present study, especially given the shorter time period, cannot be confirmed. Stress and mental fatigue signify negative aspects of affect, with the former representing a high energy state of activation and the latter a low energy state of deactivation [36]. Greater negative reactivity to stressors has been linked with risk of depression and anxiety in older people [37, 38]; therefore interventions which can reduce the affective impact of psychological stress may contribute to a reduced vulnerability to depression and anxiety. Furthermore, susceptibility to stress has been associated with a greater risk of Alzheimer's disease [39]; therefore reducing stress levels may have implications for cognitive health. Stress and mental fatigue benefits were observed for measures of current mood when measured in the home, rather than retrospective mood ratings, supporting the idea that real-time measures of mood may be more sensitive to the positive effects of nutritional interventions compared to traditional measures [14].

There are several limitations of the study which should be addressed. Mood was rated retrospectively in the laboratory (≥1 week of recall time frame) at the four-week posttreatment visit, when participants were instructed to refrain from taking the MVMH. Future studies need to fully ascertain whether abstaining from MVMH treatment on the day of follow-up assessments attenuates any mood benefits. Differences between mood states when rated in the home setting versus the laboratory also require further investigation. Mood was not rated before dose in the home, as participants were only instructed to rate their mood after consuming the multivitamin. Therefore it is not possible to determine within-day mood changes in the home setting. We utilised the in-lab assessment as our baseline, and it is possible that, due to the setting being the laboratory, mood scores may have been elevated at baseline, relative to whether assessments had taken place in a more naturalistic setting. In contrast to previous RCTs which have examined the mood effects of multivitamin supplements [32, 40], we did not examine any biochemical blood measures of vitamin status. It would have been informative to determine whether levels of B vitamins were sufficient at baseline and whether the MVMH formula was capable of increasing B vitamin levels over the four-week period, given the documented association between folate, vitamin B6, vitamin B12, and mild psychiatric symptoms [4, 12]. The supplement used in the current study also contained a range of 20 botanical extracts. The majority of herbal extracts were at a subtherapeutic dosage; however the MVMH formula contained larger extracts of the herbs Ashwagandha and Ginkgo Biloba which have been implicated in the regulation of mood [41] and flavonoid containing grape seed extract which may exert effects on the central nervous system [42]. The assessment of blood vitamin and flavonoid status at multiple time points corresponding to mood assessments may be useful to provide mechanistic insight into which components of the multivitamin may be responsible for mood alterations.

Findings from this study indicate that mobile phone devices can be used by older women to report on mood in the context of clinical trials designed to evaluate nutritional interventions. On average participants completed 7 of the 8 mood assessments indicating a satisfactory level of compliance in the home setting. In this study only a basic interface was used on the mobile phones allowing for completion of VAS ratings but not more detailed traditional mood measures. The use of a basic interface also led to clustering of scores around similar values, which would not have occurred with a touch screen application, nor on the original paper-and-pencil versions of these measures. Nevertheless due to the number of time points we were still able to identify greater stress reductions for the MVMH group, compared to placebo group, which is consistent with our findings from the acute assessment [16]. The observation of a specific multivitamin-related reduction in stress both acutely [16] and over the longer 4-week period suggests that this reduction in stress is a valid finding. Similar results of mood improvements detected using EMA measures but not standard pencil and paper measures have been observed following a mindfulness based intervention in emotionally distressed older adults [43]. These results are important as they suggest that EMA approaches could improve the detection of change in patient-reported outcomes in intervention studies when compared to standard paper-and-pencil administration [43]. It is recommended that future studies make use of smart phone applications in order to capture a broader range of mood assessments across a variety of situations (for a review of alternate technology suitable for this purpose see [14]).

In summary, the results of this investigation suggest that, over a period of four weeks, subtle changes to mood, especially stress, may not be detected when only pre- and posttreatment mood are captured. Future research is required to ascertain whether mood improvements due to multivitamin supplementation are driven by an acute effect of consuming the multivitamin [16] and therefore whether mood benefits may be diminished when the treatment is withheld.

## Figures and Tables

**Table 1:** Ingredients of Swisse Women's 50+ Ultivite formula.

Component	Daily dose
Retinyl acetate (equiv. to 2500 IU of vitamin A)	862.5 *µ*g
D-Alpha-tocopheryl acid succinate (equiv. to vitamin E 30.25 IU)	20 mg
Thiamine hydrochloride (vitamin B_1_)	30 mg
Riboflavin (vitamin B_2_)	30 mg
Nicotinamide (vitamin B_3_)	20 mg
Calcium pantothenate (vitamin B_5_) (equiv. to pantothenic acid 68.7 mg)	70 mg
Pyridoxine hydrochloride (vitamin B_6_) (equiv. to pyridoxine 20.56 mg)	30 mg
Cyanocobalamin (vitamin B_12_)	115 *µ*g
Cholecalciferol (vitamin D_3_) (equiv. to vitamin D 200 IU)	5 *µ*g
Biotin (vitamin H)	150 *µ*g
Folic acid	500 *µ*g
Calcium ascorbate dihydrate (vitamin C) (equiv. to ascorbic acid 165.3 mg)	200 mg
Phytomenadione (vitamin K)	60 *µ*g
Zinc amino acid chelate (equiv. to zinc 20 mg)	75 mg
Calcium orotate (equiv. to calcium 10 mg)	100 mg
Magnesium aspartate dihydrate (equiv. to magnesium 6.74 mg)	100 mg
Selenomethionine (equiv. to selenium 26 mcg)	65 *µ*g
Molybdenum trioxide (equiv. to molybdenum 45 *µ*g)	67.5 *µ*g
Chromium picolinate (equiv. to chromium 50 *µ*g)	402 *µ*g
Manganese amino acid chelate (equiv. to manganese 4 mg)	40 mg
Ferrous fumarate (equiv. to iron 5 mg)	16.01 mg
Copper gluconate (equiv. to copper 1.7 mg)	8.57 mg
Potassium iodide (equiv. to iodine 149.83 mcg) (equiv. to potassium 46.18 mcg)	196 *µ*g
*Lactobacillus rhamnosus *	80 million organisms
*Lactobacillus acidophilus *	80 million organisms
*Bifidobacterium longum *	35 million organisms
Citrus bioflavonoids extract	20 mg
*Vaccinium macrocarpon* fruit dry (patented cranberry Pacran)	800 mg
*Silybum marianum* dry fruit (St. Mary's thistle) (equiv. to flavanolignans calculated as silybin 17.14 mg)	1500 mg
*Ginkgo biloba* leaf dry (maidenhair tree) (equiv. to ginkgo flavonglycosides 4.8 mg and ginkgolides and bilobalide 1.2 mg)	1000 mg
*Turnera diffusa* leaf dry (damiana)	500 mg
*Scutellaria lateriflora* herb dry (skullcap)	50 mg
*Vitis vinifera* dry seed (grape seed) (equiv. to procyanidins 7.9 mg)	1000 mg
*Urtica dioica* leaf dry (nettle)	100 mg
Ubidecarenone (Coenzyme Q10) (from patented ultrasome CoQ10)	2 mg
*Cynara scolymus* leaf dry (globe artichoke)	50 mg
*Cimicifuga racemosa* root & rhizome dry (black cohosh)	200 mg
*Curcuma longa* rhizome dry (turmeric)	100 mg
*Withania somnifera* root dry (ashwagandha)	500 mg
*Crataegus monogyna* fruit dry (hawthorn)	100 mg
Silica colloidal anhydrous (equiv. to silicon 9.35 mg)	20 mg
*Bacopa monnieri* whole plant dry (*Bacopa*) (equiv. to bacosides calculated as bacoside A 1.125 mg)	50 mg
Lecithin powder-soy phosphatidylserine enriched soy (equiv. to phosphatidylserine 2 mg)	10 mg
Spearmint oil	2 mg
*Vaccinium myrtillus* fruit dry (bilberry) (equiv. to anthocyanosides 324 mcg)	100 mg
*Tagetes erecta* flower dry (marigold) (lutein esters calculated as lutein (of *Tagetes erecta*) 1 mg)	100 mg

**Table 2:** Participant demographics at baseline.

Characteristic	Multivitamin M (SD)	Placebo M (SD)
Age	64.4 (6.3)	62.8 (6.4)
Body mass index	24.4 (3.5)	26.3 (5.0)
Years of education	17 (3.4)	15.4 (3.3)^*∗*^
Employed part time (% of yes)	36%	41%
Retired (% yes)	44%	49%

^*∗*^ *p* < .05.

**Table 3:** Means and standard deviations for the mood assessments at baseline and 4 weeks after treatment.

Measure	Group		Baseline		Midpoint		Posttreatment	
		*N*	M	SD	M	SD	M	SD
*General Health Questionnaire*								
Total	Multivitamin	35	15.06	6.65	14.31	5.86	14.47	5.48
	Placebo	33	15.24	6.74	13.00	6.01	12.79	5.84
Somatic	Multivitamin	35	3.60	2.76	4.03	3.22	4.29	3.04
	Placebo	33	4.33	3.01	3.64	3.20	3.21	2.38
Anxiety	Multivitamin	35	3.57	2.76	3.42	2.52	3.20	2.58
	Placebo	33	3.48	2.58	2.97	2.56	3.00	2.52
Social dysfunction	Multivitamin	35	6.83	1.67	6.23	1.68	6.51	1.40
	Placebo	33	6.82	1.89	5.89	1.58	6.12	1.52
Depression	Multivitamin	35	1.06	2.09	0.63	1.33	0.46	1.12
	Placebo	33	0.61	1.52	0.52	1.56	0.45	1.50
*Chalder Fatigue Scale*								
Total	Multivitamin	37	16.00	4.33			13.35	3.22
	Placebo	36	14.47	3.75			13.03	3.20
Physical	Multivitamin	37	8.83	2.63			7.47	1.66
	Placebo	36	8.11	2.61			7.31	2.19
Mental	Multivitamin	37	7.05	2.17			5.62	1.40
	Placebo	36	6.36	1.53			5.72	1.39
*Hospital Anxiety and Depression Scale*								
Anxiety	Multivitamin	37	4.46	2.30			3.81	2.08
	Placebo	36	4.33	2.69			4.03	3.15
Depression	Multivitamin	37	2.16	2.10			1.35	1.57
	Placebo	36	2.23	1.75			1.42	2.00
*Perceived Stress Scale*								
	Multivitamin	37	19.05	3.21			18.70	1.90
	Placebo	35	18.40	2.08			17.74	2.00
*State-Trait Anxiety Inventory *								
State version	Multivitamin	37	32.81	10.27			28.32	6.85
	Placebo	35	30.54	9.09			27.06	9.05
*Visual Analogue Scales*								
Alertness	Multivitamin	37	68.52	17.51			72.89	16.52
	Placebo	35	70.83	16.19			75.62	17.96
Contentedness	Multivitamin	37	77.57	16.88			81.55	13.89
	Placebo	35	81.47	16.32			83.45	16.58
Calmness	Multivitamin	37	66.43	21.74			74.09	16.66
	Placebo	35	74.26	18.92			77.20	19.52
Stress	Multivitamin	37	21.76	21.61			12.38	13.63
	Placebo	35	14.77	17.81			14.09	17.15
Concentration	Multivitamin	37	65.95	28.81			72.62	20.40
	Placebo	35	61.83	26.65			66.89	29.76
Anxiety	Multivitamin	37	16.24	19.15			12.97	15.86
	Placebo	35	14.60	19.24			10.69	14.27
Mental fatigue	Multivitamin	37	23.24	24.14			18.22	20.91
	Placebo	35	23.77	21.98			19.17	19.24
Physical fatigue	Multivitamin	37	21.19	23.19			20.05	23.03
	Placebo	35	17.74	18.89			19.09	20.74

*N* = represents participants included in the pretreatment to posttreatment analysis.

**Table 4:** Fixed effects coefficients and daily odds ratios for mobile phone ratings of mood over the intervention period controlling for years of education.

Mood rating	Treatment	*Daily odds ratio*		*Odds ratio test*		*Test difference odds ratios*	
		Estimate	95% CI	*t*(71)	*p* value	*t*(71)	*p* value
Alertness	Multivitamin	1.052	(1.021, 1.084)	3.391	**.001**	1.300	.198
	Placebo	1.023	(.992, 1.055)	1.484	.142		
Contentedness	Multivitamin	1.008	(.988, 1.029)	.832	.408	.483	.631
	Placebo	1.017	(.990, 1.044)	1.241	.219		
Calmness	Multivitamin	1.044	(1.020, 1.067)	3.781	**<.001**	−.550	.584
	Placebo	1.035	(1.014, 1.056)	3.416	**.001**		
Stress	Multivitamin	.941	(.918, .965)	−4.802	**<.001**	2.820	**.006**
	Placebo	.994	(.965, 1.023)	−.442	.660		
Concentration	Multivitamin	1.022	(.995, 1.051)	1.605	.113	.220	.827
	Placebo	1.026	(1.002, 1.052)	2.133	**.036**		
Anxiety	Multivitamin	.957	(.930, .984)	−3.147	**.002**	.855	.395
	Placebo	.973	(.947, .999)	−1.630	.107		
Mental fatigue	Multivitamin	.928	(.900, .956)	−4.941	**<.001**	1.957	**.054**
	Placebo	.965	(.940, .99)	−2.802	**.007**		
Physical fatigue	Multivitamin	.963	(.935, .992)	−2.514	.014	1.682	.097
	Placebo	1.000	(.968, 1.033)	−.015	.988		
